# Monocytes and Macrophages in Pregnancy and Pre-Eclampsia

**DOI:** 10.3389/fimmu.2014.00298

**Published:** 2014-06-30

**Authors:** Marijke M. Faas, Floor Spaans, Paul De Vos

**Affiliations:** ^1^Immunoendocrinology, Department of Pathology and Medical Biology, Division of Medical Biology, University Medical Center Groningen, University of Groningen, Groningen, Netherlands

**Keywords:** pregnancy, pre-eclampsia, monocytes, macrophages, decidua, placenta

## Abstract

Preeclampsia is an important complication in pregnancy, characterized by hypertension and proteinuria in the second half of pregnancy. Generalized activation of the inflammatory response is thought to play a role in the pathogenesis of pre-eclampsia. Monocytes may play a central role in this inflammatory response. Monocytes are short lived cells that mature in the circulation and invade into tissues upon an inflammatory stimulus and develop into macrophages. Macrophages are abundantly present in the endometrium and play a role in implantation and placentation in normal pregnancy. In pre-eclampsia, these macrophages appear to be present in larger numbers and are also activated. In the present review, we focused on the role of monocytes and macrophages in the pathophysiology of pre-eclampsia.

## Introduction

Preeclampsia is one of the leading complications of pregnancy, characterized by hypertension and proteinuria and developing in the second half of pregnancy (1, 2). Preeclampsia is suggested to be a two stage disease: the first stage being poor placentation (3). The second stage is the production of pro-inflammatory factors by the diseased placenta, which activates the systemic inflammatory response, leading to the signs of pre-eclampsia (3).

During normal pregnancy, the circulation of peripheral blood through the placenta results in direct or indirect contact of maternal immune cells with the placenta. This may activate circulating immune cells, especially monocytes (4, 5). In pre-eclampsia, due to production of pro-inflammatory factors from the placenta (6–9), monocytes are even further activated and together with activation of other inflammatory cells, such as granulocytes and endothelial cells, finally induce the full blown syndrome of pre-eclampsia (3).

At the maternal–fetal interface, from the beginning of a healthy pregnancy, there is an increase of innate immune cells, such as macrophages and NK cells (10). These macrophages and NK cells may have a local immune function, however, they also appear to be important for placental development by promoting trophoblast recruitment, spiral artery remodeling, and angiogenesis (11). The present review will focus on macrophages at the maternal–fetal interface. In normal pregnancy, most of the macrophages at the maternal–fetal interface are M2 macrophages, i.e., immunomodulatory macrophages (11). In pre-eclampsia, there appear to be increased numbers of M1 macrophages, suggesting a role for these macrophages in the poor placental development in pre-eclampsia.

Monocytes and macrophages may thus play an important role in healthy pregnancy as well in the pathophysiology of pre-eclampsia. Further insight into the role of these cells in these conditions, may lead to a better understating of the inflammatory response in normal pregnancy and in pre-eclampsia. Therefore, the present paper will review the systemic and local changes in the decidua in monocytes and macrophages and their subsets during healthy human pregnancy and pre-eclampsia. Examples from animal models will also be included.

## Monocytes and Macrophages and Their Subsets

### Monocytes

Monocytes arise from precursors in the bone marrow and comprise about 5–10% of the circulating blood leukocytes. They circulate in the blood for a few days before migrating into tissues to become macrophages or dendritic cells (12). They have important functions in homeostasis, tissue repair, and inflammation (12). It has recently become clear that circulating monocytes are a heterogeneous population (12). In humans, the monocyte subsets can be distinguished based on the expression of CD14, the lipopolysaccharide (LPS) receptor. The main subset (comprising about 90–95% of the monocytes) is a subset expressing high levels of CD14, but lacking CD16 (FcγR-III) expression. Since this is the main subsets and until recently thought to be the only subset, this subset is usually called “classical subset”. The second subset of monocytes is characterized by low expression of CD14 together with CD16. This subset is often called the non-classical subset. More recently, a third, intermediate subset of monocytes has been defined, called the intermediate subset (13). This subset is characterized by high expression of CD14 in combination with expression of CD16 and is a separate subset of monocytes. It has been suggested that classical monocytes arise from the bone marrow and mature into non-classical monocytes via intermediate monocytes (13, 14). These subsets differ in many respects, including expression of adhesion molecules and chemokine receptors and function [reviewed in Ref. (12, 13)]. Classical monocytes are professional phagocytes that can generate reactive oxygen species (ROS) and produce cytokines in response to toll-like receptor dependent activation by f.i. LPS. Non-classical monocytes are weak phagocytes and do not generate ROS, but are more efficient producers of pro-inflammatory cytokines after TLR dependent activation (12). This subset has been shown to have a longer half-life and localize to both resting and inflamed tissue (12). They crawl on the luminal side of the endothelium and survey endothelial cells and tissues for damage and infection (13). Upon damage or infection, they may rapidly invade the tissue and initiate the inflammatory response (15). Non-classical monocytes have been shown to be increased in various inflammatory diseases (13, 16, 17).

### Macrophages

Macrophages are located in all body tissues, where they are important in detecting, ingesting, and processing foreign material, dead cells, and other debris (12). Monocytes are macrophage precursors (12); monocytes can be recruited into tissues, to replenish steady state macrophages or can be recruited in inflammatory conditions (12), where they mature into macrophages (or dendritic cells) (12). Macrophages play an important role in the innate and adaptive immune responses to pathogens and are important mediators of inflammatory processes (12). However, they also have anti-inflammatory properties, as they are also involved in the resolution of the inflammation (12). Indeed, several macrophage subsets with distinct functions have been described. Broadly, they can be classified into two groups: M1 or classically activated macrophages, and M2 or alternatively activated macrophages (18). These subsets differ in receptor, cytokine, and chemokine expression and in effector function (18). M1 macrophages are microbicidal and inflammatory, M2 macrophages are immunomodulatory, which can induce tolerance and the resolution of inflammation, and are only weak microbicidal (18). It has been suggested that these two populations may be extreme ends of polarization and that macrophages may actively switch their phenotype, depending on the environment (19).

There is debate on the fate of the different monocyte subsets; it is unclear whether tissue macrophages are derived from a specific monocyte subset or from either subset randomly (12). It has been suggested that classical monocytes preferentially differentiate into M1 macrophages, while the non-classical monocytes preferentially differentiate into M2 macrophages during inflammation (20). However, various studies have shown that such a strict distinction between differentiation of classical and non-classical monocytes may not be very likely and that it may depend on the model and the inflammatory stimulus whether a monocyte differentiates into an M1 or M2 macrophage (20, 21).

## Monocytes in Pregnancy

During normal pregnancy, the female immune system has to adapt to the presence of the semi-allogeneic fetus. Many changes in the peripheral circulation have been observed, both in the specific and innate immune response. In the specific immune response, a decreased Th1/Th2 ratio has been observed in both T cells (22–24) as well as in NK cells (23, 25). These changes may be associated with changes in regulatory T cells (26, 27) and Th17 cells (27). It has been suggested, that to compensate for such changes in the specific immune response, also the innate immune response has to adapt to pregnancy. This has most often been shown by increased numbers of circulating monocytes and granulocytes, resulting in increased number of total leukocytes during pregnancy (28–30). Here, we will discuss changes in monocytes during healthy pregnancy and in pre-eclampsia.

Although it has been known for a long time that leukocyte numbers increase during pregnancy, at that time this was not recognized as a sign of generalized inflammation in pregnant women. With the introduction of new techniques, most importantly, flow cytometry, function and activation status of leukocytes monocyte could be examined by measuring expression of markers of activation and production of intracellular cytokines. Moreover, the flow cytometric analysis did not require isolation of cells from whole blood, as measurements could be done in whole blood. This represents the *in vivo* situation much better, since isolation of leukocytes from blood may activate these cells (31). Using the whole blood method, Sacks et al. (32) showed phenotypical activation of monocytes during pregnancy, by showing increased expression of the activation markers CD11b, CD14, and CD64 on monocytes from pregnant women as compared with monocytes from non-pregnant women. Afterward, these results have been confirmed by others (33–35).

The monocytes are also functionally changed in pregnant women. This has, for instance, been demonstrated by measuring the production of oxygen free radicals (32), which is increased in pregnant women. Although some authors have shown increased cytokine production by non-stimulated monocytes from pregnant women vs. non-pregnant women (34), others could not confirm this finding and only observed cytokine production by stimulated monocytes (8, 30). Whether stimulated cytokine production of pregnant monocytes is increased or decreased as compared to non-pregnant women seems to depend on the stimulus. After stimulation with only LPS cytokine production by monocytes from pregnant women was decreased as compared with cytokine production by monocytes from non-pregnant women (30, 36, 37). However, after stimulation of monocytes with both LPS and IFNγ, monocytes of pregnant women showed increased cytokine production as compared with monocytes from non-pregnant women (38). Although these findings seem contradictory, they can be explained as follows: decreased cytokine production of monocytes from pregnant women following LPS stimulation is a sign of activation of monocytes, since activated monocytes become tolerant to LPS (39). IFNγ, however, abrogates LPS tolerance (40). Therefore, if LPS tolerance is abrogated by IFNγ during pregnancy, monocytes produce increased amounts of cytokines during pregnancy. The above mentioned studies have been performed in the third trimester of pregnancy and based on all above mentioned data, it is now generally accepted that monocytes are activated during pregnancy. However, little is known about monocyte activation during the course of pregnancy. However, gradually developing monocyte activation may occur during the course of pregnancy, since one paper showed progressive phenotypical activation of monocytes from the first trimester to the third trimester (34).

### Monocyte subsets in pregnancy

In the studies presented above, monocytes have been characterized by CD14 expression, indicating that mainly classical monocytes have been studied in pregnancy. Recently, we conducted a study in which we identified the three subtypes of monocytes in pregnant women (41). We showed a decreased number of classical monocytes and an increased number of intermediate monocytes in healthy pregnancy. These results are in line with the suggestion that pregnancy is an inflammatory condition, since in other inflammatory diseases, this intermediate subset has also been shown to be increased (42, 43). Our data, however, were not in line with data of Al-ofi et al. (44), who showed increased numbers of classical monocytes and decreased numbers of non-classical monocytes in pregnant vs. non-pregnant women. The reason for this difference is unclear, but may be due to differences in experimental methods. Further studies are warranted to evaluate whether the subsets respond differently to stimulation in pregnant and non-pregnant women.

### Monocytes and parturition

Parturition is associated with an inflammatory response (45). At the end of gestation, the number of leukocytes in the uterine tissue are increased (46). Also in the peripheral circulation just before delivery, further phenotypical activation of monocytes in comparison with earlier in pregnancy has been shown (47), indicating further activation of these cells just before delivery. In line with this suggestion, more recently, Vega-Sanchez et al. (48) showed differences in cytokine production of monocytes between pregnant women in labor and pregnant women not in labor. A role for activated monocytes in parturition can also be deduced from data from pre-term labor, where increased expression of activation markers by monocytes has been observed compared with healthy pregnancy (49).

### Monocytes in pre-eclampsia

It has now been well-established that during pre-eclampsia, the innate immune system is even further activated as compared with normal pregnancy (50). Activation of monocytes has been demonstrated by increased expression of inflammation associated adhesion molecules such as CD11b, ICAM-1, and CD14 (5, 32, 51, 52). However, monocytes are not only phenotypically activated, they also produced increased amounts of oxygen free radicals as compared to normal pregnancy (32) and their cytokine production also differed as compared to monocytes from normal pregnant women (38, 53–56). As for normal pregnancy, the above mentioned studies did not take into account the presence of monocyte subsets and monocytes are generally defined as CD14 positive. In our recent study, we observed decreased numbers of classical monocytes and an increased numbers of intermediate monocytes in women with pre-eclampsia as compared with normal pregnant women (41). Although Al-ofi et al. also showed decreased numbers of classical monocytes, in contrast to our study, they showed increased numbers of non-classical monocytes in pre-eclamptic women compared with healthy pregnant women (44). As explained above, this may be due to different techniques used, but may also be due to a different selection of patient groups (we exclusively included early onset pre-eclamptic women, while Al-ofi et al. included a more heterogeneous group of pre-eclamptic women).

### Possible mechanisms of monocyte activation in pregnancy and pre-eclampsia

**Figure 1:**
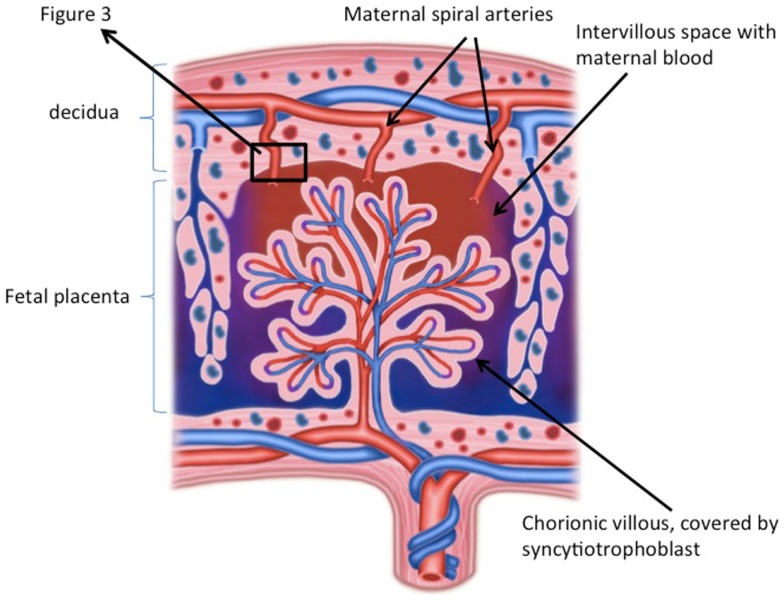
**Schematic overview of the placenta**. The placenta consists of a fetal part and a maternal part. In the fetal part of the placenta, chorionic villi, covered with syncytiotrophoblast, bath in maternal blood of the intervillous space. Direct or indirect contact (via soluble factors) of monocytes with the syncytiotrophoblast may results in monocyte activation. The maternal part of the placenta consists of decidua in which remodeled spiral arteries are present, which take maternal blood to the intervillous space. In the decidua fetal trophoblast, and maternal macrophages and NK cells are present and necessary for immune regulation and spiral artery remodeling ©ilusjessy – Fotolia.com.

The exact mechanisms involved in the activation of monocytes during pregnancy and pre-eclampsia remain unknown. The most obvious suggestion is that the placenta is involved. Peripheral monocytes circulate through the placental circulation and come into close contact with the semi-allogeneic villous syncytiotrophoblast (Figure 1). This may activate monocytes. This notion is supported by the fact that monocytes become activated during their passage through the placenta (5). It is, however, unsure whether this activation of monocytes occurs due to direct contact, since several soluble placental products, such as cytokines (57), placental microparticles (58), fetal DNA (59), released into the maternal circulation, may also activate monocytes (60).

Many factors may be involved in further activation of monocytes during pre-eclampsia. Factors may be derived from the stressed placenta, such as anti-angiogenic factors (61), placental microparticles (62), or ATP (9), which are released at increased amounts from the pre-eclamptic placenta. These factors may activate the monocytes. Also upregulation of various pro-inflammatory cytokines, such as TNFα, IL-1β, IL-18, in the placenta of pre-eclamptic women has been observed (63–65). On the other hand, decreased levels of the anti-inflammatory cytokine IL-10 have been observed in the placenta of pre-eclamptic women (66, 67). These increased levels of pro-inflammatory cytokines in the pre-eclamptic placenta may be responsible for the increased circulating levels of these cytokines in pre-eclamptic patients (68, 69). These cytokines may also activate the monocytes. Since monocytes themselves are potent producers of cytokines, the activation of monocyte by placental factors and cytokines may in turn result in a vicious circle of monocytes activation and cytokine production leading to persistent increased monocyte activation in pre-eclampsia.

It appears to be important for induction of pre-eclamptic signs how monocytes are activated. In pregnant rats, hypertension and proteinuria can only be induced after infusion with *E coli* LPS (70), not after infusion of LPS from *Porphyromonas gingivalis* (71), despite the fact that monocytes are activated by this LPS (72). This may explain why certain infections, such as urinary tract infections or periodontitis, may increase the risk of pre-eclampsia, while other infections, such as CMV or malaria do not increase the risk for pre-eclampsia (73). Apparently, the immune response, and specifically monocyte activation is different in different infections. Differences may amongst others relate to differences in cytokine production between states of monocytes activation, since we have previously shown that activation of monocytes with *E coli* LPS or *P. gingivalis* LPS resulted in different cytokine production (36).

### Monocytes during pregnancy and experimental pre-eclampsia in animals

Although it is now generally accepted that during pregnancy monocytes are activated and that they are even further activated during pre-eclampsia, whether this is the cause or consequence of pre-eclampsia still remains to be shown. It is difficult to study the role of monocytes in pregnancy and pre-eclampsia in human subjects. Therefore, animal models are needed. A good animal model to study innate immune responses in pregnancy is the rat. Although not completely similar, like humans, rats have a hemochorial placenta, showing deep trophoblast invasion into the uterine wall (74) indicating that fetal–maternal interactions may be similar in rat and human pregnancy. Therefore pregnancy-induced changes in the immune response may also be similar to human pregnancy. Indeed, similar phenotypical and functional activation of monocytes during the course of pregnancy have been observed in rats as compared with humans (75, 76). Moreover, in accordance with human pregnancy, we found decreased numbers of classical monocytes and increased numbers of non-classical monocytes during pregnancy in this species (41).

Various rat models have suggested that activation of monocytes, by LPS, ATP, or TNFα during pregnancy, induced pre-eclampsia-like signs (70, 77, 78). Interestingly, such pre-eclampsia-like syndromes were only induced in pregnant rats, not in non-pregnant rats (70, 77). The pathophysiology of the LPS and ATP induced pre-eclampsia was characterized by a pregnancy-specific inflammatory response, characterized by persistent general (75, 76, 79) and glomerular (79, 80) inflammation, in which monocytes play a major role. In the ATP model, we have shown that, similar to human pre-eclampsia, non-classical monocytes are increased and activated by ATP, suggesting an important role for this subset in pre-eclampsia. Together, these animal studies support the hypothesis that activation of monocytes in pregnancy may result in pre-eclampsia-like signs, such as hypertension and proteinuria.

**Figure 2:**
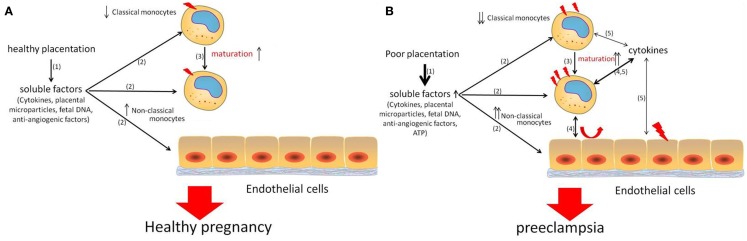
**Schematic overview of the role of monocytes during healthy pregnancy (A) and pre-eclampsia (B)**. During healthy pregnancy, placental factors (1) activate monocytes (2) and may affect endothelial cells (2) and induce increased maturation of classical monocytes toward non-classical monocytes (3). During pre-eclampsia, more and other soluble factors are produced from the stressed placenta (1), resulting in further activation of monocytes and endothelial cells (2) and further maturation of classical monocytes toward non-classical monocytes (3). Numbers non-classical monocytes are increased and they may play an important role in this inflammatory process, since they are known to produce increased numbers of cytokines upon activation (4). These cytokines further activate the monocytes (5) as well as endothelial cells (5). This vicious circle of activation of monocytes and endothelial cells finally results in the symptoms of pre-eclampsia, such as hypertension and proteinuria.

Based on the above data on monocytes during pregnancy and pre-eclampsia, we suggest that factors that arise from the healthy placenta during pregnancy induce phenotypical activation of monocytes and induce increased maturation toward non-classical monocytes. These factors may also affect endothelial cells directly (Figure 2A). During pre-eclampsia, the stressed placenta starts to produce various pro-inflammatory factors, which further activate the monocytes and further increased monocyte maturation toward non-classical monocytes. Monocyte activation results in monocyte cytokine production. Via a vicious circle, these cytokines may further activate the monocytes themselves as well as the endothelial cells, finally resulting in the signs of pre-eclampsia, such as proteinuria and hypertension (Figure 2B).

## Decidual Macrophages

Macrophages are already present in the non-pregnant endometrium, although in low numbers (81). Since their numbers fluctuate during the menstrual cycle (81, 82), it seems likely that these are under hormonal control (83). After fertilization, the number of uterine macrophages increase, due to expression of various chemokines (84) and during pregnancy macrophages are abundantly present in the decidua at all times of pregnancy (85). During pregnancy, they comprise about 20–30% of all decidual leukocytes (86, 87). The number of decidual macrophages may vary with gestational age being highest in the first and second trimester (88). Macrophages in the decidua are usually associated with spiral arteries and glands as well as with extravillous trophoblast (86, 89), but are also found in the myometrium (85). When the presence of macrophages in the decidua was first discovered, it was suggested that these cells were recruited as the result of an immune response to the semi-allogeneic fetus (90). However, it is now generally accepted that macrophages, and other immune cells present in the decidua, are necessary for successful implantation (85). Various studies have focused on specific functions of macrophages in the decidua and it has been suggested that the decidual macrophages have various roles during pregnancy, mainly in placentation (91), but they may also play a role in protecting the fetus against intrauterine infection (92).

### Decidual macrophages in early pregnancy

Most of the studies on macrophages in the decidua have been performed in early pregnancy. At this time of pregnancy, macrophages are located near the spiral arteries during trophoblast invasion and spiral artery remodeling (86, 89). The role of macrophages in spiral artery remodeling was further emphasized by the fact that they are present even before the presence of extravillous trophoblast (93). At that time, disruption and disorganization of vascular smooth muscle cells and endothelial cells was also observed (93). This suggests that macrophages may be important in the very early phases of spiral artery remodeling, preparing the spiral arteries for further remodeling by trophoblast cells (93). Their suggested role in vascular remodeling is in accordance with the findings of production of factors associated with angiogenesis and tissue remodeling by these cells (94, 95). Indeed macrophages, which were MMP 9 positive, and which were shown to have phagocytotic capacities were found to infiltrate spiral arteries during remodeling (96). Macrophages have also been shown to be important for clearance of apoptotic cells in the decidua (97). Apoptosis is an important process during spiral artery remodeling and trophoblast invasion. During these processes, apoptotic trophoblast cells (98) as well apoptotic cells in the vascular wall that is being remodeled have been observed (93). By engulfment of the apoptotic cells, macrophages prevent the release of pro-inflammatory substances from the apoptotic cells into the decidua [reviewed in Ref. (97)].

Decidual macrophages have mainly been classified as M2-like macrophages, i.e., immunomodulatory macrophages (99). Although they express many markers of M2 macrophages, such as CD206, CD163, and DC-sign (100–102), they appeared not to be typical M2 macrophages, since they are not induced by Th2 cytokines, such as IL4, but by M-CSF and IL-10 (102). These data are in line with the abundant presence of M-CSF and IL-10 in the decidua (103–105). The M2 phenotype is most likely due to hypermethylation of genes encoding markers of classical activation and hypomethylation of genes encoding markers for non-classical activation (106). Next to the typical M2 cytokine gene expression, these decidual macrophages also showed gene expression for inflammatory cytokines such as IL-6 and TNFα (102, 107). The production of pro-inflammatory cytokines by decidual macrophages may also be explained by the presence of two macrophage subpopulations in the early decidua (107). One of these subsets may be a more pro-inflammatory subset, since this subset expressed genes associated with inflammation. The other subset, which was higher in number, expressed genes associated with extracellular matrix formation, networking, communication, and growth (107).

Apart from the putative role of M-CSF and IL-10 in induction of M2 macrophages in the decidua, other factors have also been suggested to be involved in inducing/maintaining the M2 phenotype in decidual macrophages. Decidual macrophages have been shown to express inhibitory receptors immunoglobulin like transcript (ILT)2 and ILT4 (108). These receptors can bind to HLA-G expressed on invading extravillous trophoblast (108), after which a negative signal is delivered to the macrophages, resulting in tolerance to the trophoblast and the induction of anti-inflammatory cytokines. It has also been suggested that the engulfment of the apoptotic cells induced an immunosuppressive and anti-inflammatory phenotype of the macrophages (97). Not only the phagocytosis of apoptotic cells, but also the phagocytosis of trophoblast cell debris at the maternal–fetal interface may be associated with the M2 phenotype of macrophages (109–111). In addition, as it has been suggested that non-classical monocytes preferentially differentiate into M2 macrophages (20), it may be speculated that the increased numbers of non-classical monocytes in the circulation during pregnancy (41), results in increased invasion of these cells into the decidua to become M2 macrophages.

### Decidual macrophages in late pregnancy

Macrophages are present in the decidua throughout pregnancy until the end of pregnancy, although their numbers may decrease at the end of pregnancy (88). The exact role of decidual macrophages at the end of pregnancy remains to be established, it seems, however, likely that they are still involved in immunoregulation and clearance of apoptotic cells. Indeed, many of the macrophages present in the decidua at the end of pregnancy, appeared to be M2 macrophages (112). The potential protective effect of M2 macrophages for the fetus was recently shown by van Schonkeren et al., who showed the presence of an inflammatory lesion in placentae from women who underwent egg donation (113). This lesion consisted of maternal cells, expressing high levels of CD14 and CD163, suggesting the presence of M2 macrophages. These lesions appeared to protect against pre-eclampsia (113).

### Decidual macrophages in pre-eclampsia

Preeclampsia is associated with defective trophoblast invasion and spiral artery remodeling: while in healthy pregnancy, spiral artery remodeling extends into the myometrium, in pre-eclampsia, spiral artery remodeling can only be found in the decidua (3). Unfortunately, not very many studies focused on macrophages in the decidua in pre-eclampsia. Most of the studies in pre-eclampsia were obviously performed after delivery of the placenta. Some of the studies reported decreased numbers of macrophages in the decidua of pre-eclamptic patients (114, 115). Most of the studies, however, found increased numbers of macrophages in pre-eclamptic patients (112, 116–118). These data may not necessarily be conflicting, since not only different methods were used (Williams and Burk performed a flow cytometric study, while the other studies were immunohistochemical studies), also different antibodies were used. Increased numbers of macrophages in the decidua of pre-eclamptic patients appears to be in line with increased presence of macrophage chemotactic factors, such as M-CSF, IL-8, and MCP-1 (119–121) in pre-eclamptic patients. Not only numbers of macrophages were found to be different in pre-eclamptic patients, macrophages may also be differently activated in pre-eclampsia (120–122). This may be in line with the presence of increased pro-inflammatory cytokines (63–65) and decreased anti-inflammatory cytokines in the placenta of pre-eclamptic women (66, 67). More recently, it has been shown that in the decidua of pre-eclamptic women decreased numbers of M2 macrophages are present (112). Differences in macrophage numbers may be regional, since increased numbers of macrophages were found around the spiral arteries of pre-eclamptic patients (120, 121). The presence of macrophages in the spiral arteries may be associated with the development of acute artherosis (120). Acute artherosis is a process mainly seen in poorly remodeled spiral arteries at the end of pregnancy, characterized by the presence of subendothelial CD68 positive foam cells (123). Its presence is associated with adverse fetal and maternal outcome (124).

The question remains whether the increased presence of macrophages in the decidua of pre-eclamptic women is the cause or the result of pre-eclampsia. This question is difficult to answer, due to the difficulties of obtaining material from early decidua of women who later in pregnancy developed pre-eclampsia. However, recently we have shown that in early decidua from women who later developed pregnancy-induced hypertension (PIH) (including pre-eclampsia) CD68 mRNA expression was increased (125), suggesting increased numbers of macrophages in the early decidua of women who later develop hypertension in pregnancy. Moreover, the CD206/CD68 mRNA ratio was decreased in PIH women, suggesting that decreased numbers of M2 macrophages are present in the decidua of women who later develop pregnancy-induced hypertension (125). The increased numbers of macrophages, with decreased numbers of M2 macrophages may thus already be present before the onset pre-eclampsia and therefore suggest a role for macrophages in defective trophoblast invasion and spiral artery remodeling. Recent *in vitro* data showed that macrophages migrate toward invading trophoblast (126), while other groups have shown that activated macrophages *in vitro* are able to inhibit trophoblast invasion and spiral artery remodeling (127, 128). *In vivo*, data have shown that there is a reciprocal presence of trophoblast cells and macrophages in the spiral arteries of both healthy and pre-eclamptic women (121). Therefore the increased numbers of macrophages in and around spiral arteries of pre-eclamptic women (121) may inhibit spiral artery remodeling.

Since it is difficult to study the role of macrophages in pre-eclampsia in humans, animal models may help in understanding critical questions. Studying whether trophoblast invasion and spiral artery remodeling is associated with macrophages in animal models for pre-eclampsia may shed light on the question whether increased numbers of macrophages in the decidua are the cause or the result of pre-eclampsia. In an animal model for pre-eclampsia induced by multiple doses of LPS in pregnant rats, decreased trophoblast invasion and spiral artery remodeling after LPS was associated with increased numbers of macrophages. We studied this subject and showed increased invasion of activated macrophages in the mesometrial triangle (the equivalent of the placental bed in humans) before defective trophoblast invasion and spiral artery remodeling (129). This appears to be in line with the sparse human data and suggests a role for activated macrophages in the pathophysiology of pre-eclampsia.

**Figure 3:**
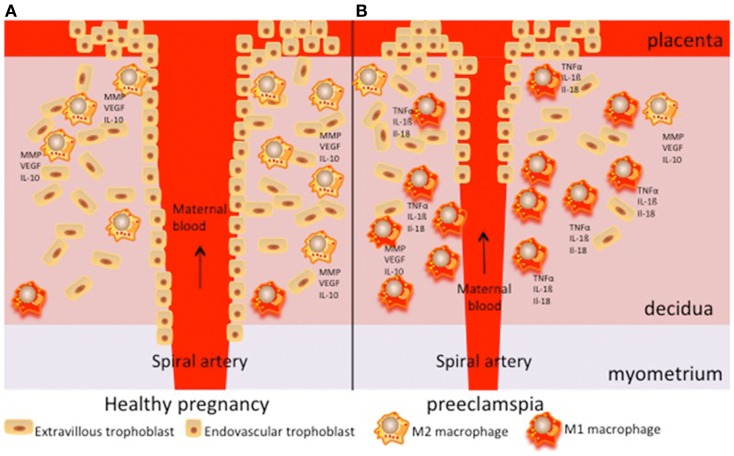
**Schematic overview of the role of decidual macrophages in pregnancy (A) and pre-eclampsia (B)**. During normal pregnancy, M2-like macrophages are present around spiral arteries and play a role in remodeling of these arteries by producing various factors associated with angiogenesis and tissue remodeling (such as MMP and VEGF). They also play a role in immunomodulation, for instance by producing IL-10. During pre-eclampsia, increased numbers of M1-like macrophages are found. They may produce pro-inflammatory cytokines, such as TNFα, IL-1β, or IL-18.

M2-like macrophages are thus abundantly present in the decidua of healthy pregnant women. They are observed in the presence of spiral arteries and extravillous trophoblast cells and may play a role in spiral artery remodeling by producing factors associated with angiogenesis and tissue remodeling, such as MMPs and VEGF (Figure 3A). During pre-eclampsia, increased numbers of decidual macrophages are observed, which may be of the M1 phenotype and therefore produce pro-inflammatory cytokines (Figure 3B). These activated macrophages may affect spiral arteries and may induce acute artherosis, affecting the placental blood circulation.

## Summary

**Figure 4:**
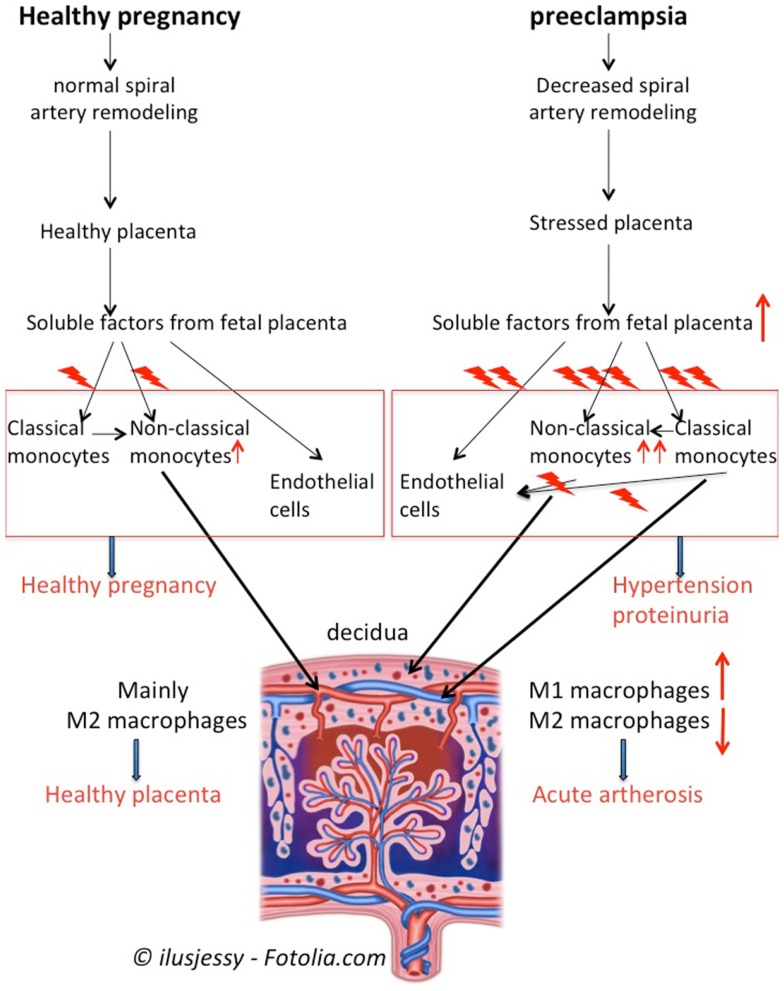
**Summary of monocytes and macrophages in pregnancy and pre-eclampsia**. In healthy pregnancy, soluble factors from the villous trophoblast activate circulating monocytes, induce maturation of classical monocytes toward non-classical monocytes and affect endothelial cells. Non-classical monocytes will invade into the decidua to become M2-like macrophages to support healthy placentation and immunomodulation. During pre-eclampsia, decreased remodeling of the spiral arteries will results in a stressed placenta, which produces increased amounts or different soluble factors as compared with healthy pregnancy. The soluble factors will further activate the monocytes, induce further maturation of classical monocytes toward non-classical monocytes and activate endothelial cells. Activated monocytes, by f.i. producing cytokines, further affect monocytes and endothelial cells. This vicious circle of monocyte and endothelial cell activation results in the maternal symptoms of pre-eclampsia, i.e., hypertension and proteinuria. Moreover, activated classical and non-classical monocytes may invade into the decidua to develop into M1-like and M2-like macrophages, resulting in increased numbers of M1-like macrophages in the pre-eclamptic decidua. The M1-like macrophages may affect the spiral arteries resulting in f.i. acute atherosis, thereby further affecting the placental blood circulation.

Monocytes and macrophages play important roles in pregnancy and pre-eclampsia. Monocyte activation and increased numbers of non-classical monocytes, is important for normal pregnancy. Monocyte derived macrophages, especially M2-like macrophages (which may be derived from non-classical monocytes) in the decidua in healthy pregnancy play an important role in blastocyst implantation, trophoblast invasion, and spiral artery remodeling as well as in defense against infection and in immunomodulation (Figure 4). During pre-eclampsia, decreased spiral artery remodeling results in increased production of soluble factors (or different factors), inducing further activation of both classical and non-classical monocytes and further maturation toward non-classical monocytes. These placental factors as well as the activated monocytes also induce activation of endothelial cells. Activated monocytes (both classical and non-classical monocytes) may invade into the decidua, resulting in increased numbers of M1-like macrophages in the decidua of pre-eclamptic women (Figure 4). The M1-like macrophages may affect the spiral arteries, by for instance inducing acute artherosis. This may further affect the placental blood circulation and stress the placenta.

Unfortunately, most studies on monocytes and macrophages in pre-eclampsia have been performed during pre-eclampsia. Although we do believe that monocytes and decidual macrophages do play a role in inducing the maternal symptoms of pre-eclampsia, it is relatively unknown whether monocytes and decidual macrophages do also play a role in the aberrant spiral artery remodeling early in pregnancy. The question thus remains as to what induces the aberrant spiral artery remodeling? Future studies should therefore not only focus on the three monocyte subsets in pregnancy and pre-eclampsia, but also on the relationship between the circulating monocyte subsets and macrophages in the decidua. Moreover, since data on macrophages in the decidua in and before pre-eclampsia are relatively scarce future studies should therefore also focus on macrophage function and phenotype in and before pre-eclampsia.

## Conflict of Interest Statement

The authors declare that the research was conducted in the absence of any commercial or financial relationships that could be construed as a potential conflict of interest.
